# Imputing Alzheimer's disease phenotype in individuals with mild cognitive impairment for inclusion in gene discovery tests

**DOI:** 10.1002/alz70855_107001

**Published:** 2025-12-25

**Authors:** Eden R. Martin, Anthony J Griswold, Farid Rajabli, Tianjie Gu, Charles G. Golightly, Patrice G Whitehead, Kara L Hamilton‐Nelson, Larry D Adams, Jose J. Sanchez, Vanessa C. Rodriguez, Pedro R Mena, Katrina Celis, Takiyah D. Starks, Concepcion Silva, Gary W Beecham, William S Bush, Christiane Reitz, Katalina F. McInerney, Michael L Cuccaro, Jeffery M Vance, Briseida E. Feliciano‐Astacio, Goldie S Byrd, Jonathan L Haines, Margaret Pericak‐Vance

**Affiliations:** ^1^ Dr. John T. Macdonald Foundation Department of Human Genetics, University of Miami Miller School of Medicine, Miami, FL, USA; ^2^ John P. Hussman Institute for Human Genomics, University of Miami Miller School of Medicine, Miami, FL, USA; ^3^ University of Miami Miller School of Medicine, Miami, FL, USA; ^4^ Maya Angelou Center for Health Equity, Wake Forest University School of Medicine, Winston‐Salem, NC, USA; ^5^ Department of Biostatistics and Data Science, Wake Forest School University of Medicine, Winston‐Salem, NC, USA; ^6^ Department of Population and Quantitative Health Sciences, School of Medicine, Case Western Reserve University, Cleveland, OH, USA; ^7^ Cleveland Institute for Computational Biology, Department of Population & Quantitative Health Sciences, School of Medicine, Case Western Reserve University, Cleveland, OH, USA; ^8^ Columbia University Irving Medical Center, New York, NY, USA; ^9^ Department of Neurology, University of Miami Miller School of Medicine, Miami, FL, USA; ^10^ Universidad Central del Caribe, Bayamon, PR, USA; ^11^ Wake Forest University School of Medicine, Winston‐Salem, NC, USA; ^12^ Department of Population and Quantitative Health Sciences, Institute for Computational Biology, Case Western Reserve University, Cleveland, OH, USA

## Abstract

**Background:**

Genetic association studies of Alzheimer's disease (AD) typically compare cognitively unimpaired (CU) controls to clinically diagnosed AD cases, excluding individuals with mild cognitive impairment (MCI) due to uncertainty about progression. We hypothesize that estimating the probability of MCI individuals developing AD could enhance case‐control analyses and improve detection of genetic associations.

**Method:**

Given a dataset of AD cases, CU controls, and MCI individuals, we propose a multi‐step approach: (1) Use stepwise logistic regression with AD and CU individuals (excluding MCI) to identify a “best model” based on non‐genetic covariates, e.g., clinical, demographic, and biomarker data; (2) Fit this model to estimate the probability (*p_i_*) of AD for each MCI individual; (3) Incorporate MCI individuals as “cases” in a genetic association test using two possible approaches. The first reclassifies MCI individuals with *p_i_* > *t* as cases and includes them in an association test statistic. The second resamples MCI individuals as cases probabilistically based on *p_i_* to generate an empirical distribution of test statistics. As a proof of principle, we applied this framework to assess the association of the APOE‐e4 allele with AD in 1420 individuals (448 AD cases, 714 CU controls, 258 MCI).

**Results:**

As a benchmark, testing APOE‐e4 using only AD cases and CU controls yielded an odds ratio (OR) of 3.25 and test statistic Z = 9.088. Including all MCI individuals as cases reduced the effect and statistic (OR=1.97;Z=6.628). Our imputation procedure identified a “best model” incorporating cohort, sex, age, pTau‐181, memory box score from Clinical Dementia Rating, and interactions between pTau‐181, age, and sex. The threshold model with *t* between 0.6 and 1 slightly improved the test statistic compared to using cases and controls alone, e.g., *t* = 0.8 adds 24 MCI individuals to cases and yields OR=3.22;Z=9.174. The resampling approach performed better than including all MCI individuals but not as well as the threshold method.

**Conclusion:**

Clinical, demographic, and biomarker data can be used to impute “caseness” for MCI individuals in genetic association tests. Even a small number of imputed MCI cases improved test statistics, suggesting greater benefits in larger datasets.

